# Sexually Transmitted Disease Partner Notification among African-American, Adolescent Women

**DOI:** 10.1155/2014/619632

**Published:** 2014-12-25

**Authors:** Anna Buchsbaum, Maria F. Gallo, Maura K. Whiteman, Carrie Cwiak, Peggy Goedken, Joan Marie Kraft, Denise J. Jamieson, Melissa Kottke

**Affiliations:** ^1^Division of Family Planning, Department of Gynecology and Obstetrics, Emory University, 49 Jesse Hill Jr., Drive SE, Atlanta, GA 30303, USA; ^2^Division of Epidemiology, College of Public Health, Ohio State University, 324 Cunz Hall, 1841 Neil Avenue, Columbus, OH 43210, USA; ^3^Division of Reproductive Health, Centers for Disease Control and Prevention, 4770 Buford Highway, Mail Stop K-34, Atlanta, GA 30341-3724, USA

## Abstract

*Objective*. To better understand preferences and practices regarding partner notification of sexually transmitted infection (STI) among female, African-American adolescents. *Methods*. Participants completed a questionnaire and STI testing at baseline. Those diagnosed with Chlamydia or gonorrhea were recruited for a follow-up study, involving another questionnaire and repeat STI testing after three months. *Results*. At baseline, most participants (85.1%) preferred to tell their partner about an STI diagnosis themselves instead of having a health care provider inform him, and 71.0% preferred to bring their partner for clinic treatment instead of giving him pills or a prescription. Two-thirds of participants were classified as having high self-efficacy for partner notification of a positive STI diagnosis. In the multivariable analysis, older participants and those with fewer lifetime sexual partners were more likely to have high self-efficacy. Ninety-three participants (26.6%) had Chlamydia or gonorrhea and, of this subset, 55 participated in the follow-up study. Most adolescents in the follow-up study (76.4%) notified their partner about their infection. *Conclusion*. Although participants were willing to use most methods of partner notification, most preferred to tell partners themselves and few preferred expedited partner therapy. Traditional methods for partner notification and treatment may not be adequate for all adolescents in this population.

## 1. Introduction

African-American adolescent women in the southern United States have a disproportionately high burden of Chlamydia and gonorrhea infection compared to adolescents of different race, ethnicity, or geographic location [1]. Repeat cases are significantly more common in adolescents than in their older counterparts [2], which could contribute to the high prevalence of disease among this population. For example, in 1993–1998 in Washington State, 17% of women 10–19 years of age had repeat Chlamydia within two years compared to 4–10% of women 20–44 years of age [3]. To reduce reinfection, partners of infected patients must be treated for the appropriate sexually transmitted infection (STI), which first requires their notification. Traditionally, partners of patients diagnosed with an STI are notified and referred to medical treatment by the infected person (patient referral) or by a medical provider (provider referral) [4].

While many strategies for improving partner notification have been studied, no single method has emerged as clearly superior [5, 6]. Furthermore, limited evidence is available on preferences and practices among adolescent women specifically. Adolescence often is marked by profound changes in understanding and exploring of sexual relationships, and partnerships among youth can be more transitory or undefined than those among adults. Because of the nature of their developmental stages, communication skills, knowledge gaps, and type of relationships, adolescents may require different strategies for partner notification. Persistently high STI rates suggest that traditional management schemes may be insufficient. While 61%–75% of adolescent women report notifying their partners of a positive STI diagnosis, only 25–79% of this subset also stated that their partners were treated [7–10]. This study aimed to (1) describe preferences for various partner notification and treatment strategies among a sample of sexually active, African-American, female adolescents in Atlanta, Georgia; (2) identify individual, relationship, and other psychosocial factors associated with high self-efficacy for partner notification of a potential STI diagnosis; (3) determine the frequency of partner notification among those testing positive for Chlamydia or gonorrhea; and (4) measure repeat STI diagnosis among this high-risk population of female adolescents.

## 2. Materials and Methods

The study was conducted among a convenience sample of young African-American women attending an urban clinic in the southern USA. The study clinic was supported by the Title X Family Planning Grant to provide family planning, STI, and preventive health services to individuals ≤19 years of age. Participants had to meet the following eligibility criteria: they should be female, English speaking, born in the USA, 14–19 years of age, and sexually active (defined as vaginal intercourse in the past six months) with a male partner; they should self-identify as African-American, present to the clinic for care, and be willing to provide written consent if age ≥ 18 or assent if they are 14–17 years old.

All female patients presenting from April 2012 through September 2012 were invited to participate, and eligible patients providing consent or assent were enrolled. The study included the administration of a structured questionnaire and collection of urine samples for testing for Chlamydia and gonorrhea with APTIMA Combo2 assay (Gen-Probe, Inc., San Diego, CA). Participants with a positive diagnosis were treated per clinic standard of care, which included directly observed therapy and counseling to return for repeat testing three months after treatment [11]. Standard counseling involved encouraging their partners to be tested and treated and reviewing STI prevention strategies, including abstinence and condom use. The study clinic offered partner treatment but not expedited partner therapy (EPT) (defined as providing infected patients with medication or a prescription for medication to give directly to their partners) as the latter practice was not explicitly legal in this setting.

Using a tablet computer, participants completed an audio computer-assisted self-interviewing (ACASI) questionnaire on demographics; relationship characteristics; contraceptive, pregnancy, and STI histories; and communication. Then given a theoretical situation of testing positive for an STI, participants were asked to answer questions on preferences for partner notification and treatment and self-efficacy regarding partner notification. The latter was assessed by expanding a three-item scale used by Fortenberry and colleagues [9]. That is, we asked six questions using a Likert-type scale with four possible responses (*very sure, sure, unsure*, and* very unsure*) to measure participant self-efficacy regarding her ability to tell her partner of an STI, ask her partner to get STI testing, ask her partner to get STI treatment, abstain from sex with her partner until he obtained STI treatment, ask her partner whether he was tested, and ask her partner whether he was treated. Because each of these six measures is important for preventing reinfection,* high self-efficacy* was defined as answering all six questions with* very sure* or* sure*. This derived variable had high internal consistency (Cronbach's coefficient alpha = 0.85).

Participants who tested positive for Chlamydia or gonorrhea at baseline were eligible to participate in a follow-up study, which required a return to the clinic in three months to complete another structured questionnaire using ACASI and to provide, per clinic standard of care, a urine sample for repeat STI testing. Each participant was questioned on whether she had notified her most recent partner (identified at the baseline visit) of her positive STI test; reasons for or against this notification; type of information relayed; perceived partner reactions; her preferences for partner notification and treatment strategies; and whether she believed her partner was treated. To improve recall, participants were provided calendars and were reminded of the initials of their most recent sexual partner from their baseline questionnaire. Participants who tested positive for a repeat infection were treated per standards of the clinic and the Centers for Disease Control and Prevention (CDC) [11]. We attempted to contact all eligible participants who did not return for the follow-up study to request that they complete the follow-up questionnaire via telephone. Institutional review boards at Emory University School of Medicine and the CDC approved the research.

Baseline characteristics and preferences for partner notification and treatment strategies were evaluated for differences by baseline STI status (as a measure of risk behavior) using chi-squared tests. Logistic regression was used to identify correlates of high self-efficacy for partner notification at baseline. Given the lack of data on correlates of this self-efficacy, we instead focused on factors that have been identified in the literature as correlates of partner notification: age, STI history, pregnancy history with current partner, age at first sex, lifetime number of sex partners, relationship length and type, and agreeing to monogamy with partner. Variables were first assessed in bivariable models and those with evidence of an unadjusted association with high self-efficacy (*P* value < 0.10 from the bivariable model) were retained in the final multivariable model. Differences between participants who had an STI and completed the follow-up study versus participants who had an STI and did not participate in the follow-up were evaluated with chi-squared tests. The small sample size in the follow-up study precluded carrying out planned analyses to identify correlates of partner notification among those testing positive for STI at baseline. Instead, we only evaluated the unadjusted association between high self-efficacy and self-reported partner notification.

## 3. Results

**Figure 1:**
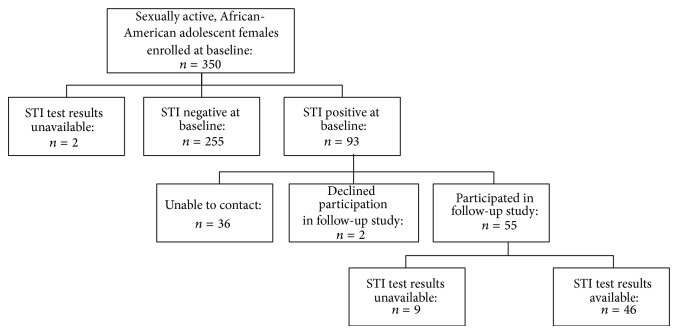
Disposition of participants.

We enrolled 350 female adolescents in the study (Figure 1). Of the 348 participants who had STI tests performed, 26.7% (*n* = 93) tested positive for ≥1 study STI: 24.1% had Chlamydia (*n* = 84), 5.2% had gonorrhea (*n* = 18), and 2.6% had both infections (*n* = 9). Nearly all of the participants (92.3%) were students. Most participants (65.1%) were ≥17 years old and had public or private health insurance (61.7%) (Table 1). Approximately half (50.3%) were currently sexually active with a* serious* boyfriend at baseline. Compared with those who tested negative at baseline, those with Chlamydia or gonorrhea were more likely to be new clinic patients (*P* < 0.01) and to think that they might have an STI (*P* < 0.01).

If they were to hypothetically test positive for an STI, most participants reported a preference for informing their partner themselves (85.1%) instead of having a health care provider notify him (13.5%) or not telling him at all (1.4%) (Table 2). When asked about their preferred method for partner treatment for an STI, 71.0% reported preferring to bring him to clinic. Fewer participants preferred to tell him to get tested and treated (17.6%) or to give him medication or a prescription for medication (11.2%). Participants were asked in a series of questions whether, if provided by the clinic, they would give the following items to their partner: antibiotic prescription, antibiotic pills, referral sheet, information pamphlet, or a clinic appointment. For each question, >90% of participants reported willingness to give the item. None of the participant preferences differed significantly by baseline STI status.

Most participants answered* sure* or* very sure* to the six questions on self-efficacy for partner notification of a positive STI diagnosis, which led to 66.3% being classified as having overall high self-efficacy. Five variables were associated with this high self-efficacy in the bivariable analysis: older age, ever pregnancy with current partner, fewer lifetime sex partners, most recent sex partner being a serious boyfriend, and having agreed to monogamy with their most recent sex partner (Table 3). Only two factors were statistically significant in the multivariable analysis. Older adolescents (17–19 years of age) were more likely to be classified as having high self-efficacy (adjusted odds ratio (OR), 2.0; 95% confidence interval (CI), 1.3–3.3) compared to younger adolescents (14–16 years). Also, those with ≤3 lifetime sex partners were more likely to have high self-efficacy (OR, 2.0; 95% CI, 1.2–3.2) compared to those with >3 lifetime partners.

Ninety-three participants tested positive for an STI at baseline and therefore were eligible for the follow-up study (Figure 1). Two declined to participate, and 36 could not be contacted because of inaccurate or changed telephone numbers. Thus, 55 (59%) enrolled in the follow-up study. Nine participants answered the follow-up questionnaire by telephone and, consequently, did not have follow-up STI testing performed. Compared to the 38 adolescents who had an STI and did not participate in the follow-up study, those who had an STI and participated in the follow-up study were more likely to be <15 years old at first sex (31.6% versus 58.2%, resp.; *P* = 0.01), were more likely have ≥3 lifetime sexual partners (36.8% versus 58.2%, resp.; *P* = 0.04), and were less likely to have agreed to monogamy with their sexual partner (91.9% versus 74.6%, resp.; *P* = 0.04).

At follow-up, most participants (*n* = 42; 76.4%) reported having told their most recent sexual partner of their positive STI diagnosis (Table 4). For 23 of these participants, this was a “serious” boyfriend. Participants described informing their partners in person (*n* = 24), by telephone (*n* = 13), by text (*n* = 4), or with a referral sheet (*n* = 1); none reported using provider referral. About half of participants at follow-up reported that their partner was tested (*n* = 28) and treated for an STI (*n* = 25). High self-efficacy at baseline for notifying a partner of an STI diagnosis was associated with subsequent partner notification (OR 4.5, 95% CI 1.2–17.2). Among those who informed their partner, the main reason cited was wanting him to know that he had infected her (*n* = 15) followed by not wanting him to reinfect her (*n* = 10) or infect others (*n* = 11). Other reasons included wanting him to know that he was possibly infected (*n* = 3), being instructed by their health care provider to do so (*n* = 2), or feeling like it was the right thing to do (*n* = 1). Among the minority who did not inform their partner of their infection, their reasons for failing to tell included not being aware of their STI (*n* = 3), no longer dating him (*n* = 2), believing that they were infected by a different partner (*n* = 2), thinking that he would become very upset (*n* = 2), fearing physical abuse (*n* = 1), not knowing where to find him (*n* = 1), thinking someone else already informed him (*n* = 1), or already having been told by him that he had an STI (*n* = 1).

Overall, many participants reported that their partner accepted the news well (*n* = 19) or questioned them to learn more (*n* = 17) (Table 4). However, some reported a negative response from their partner including his becoming upset (*n* = 11), accusing her of having sex with another person (*n* = 10), or threatening her with physical abuse (*n* = 1). Participants reported that they provided their partner with information on his need to get tested (*n* = 41) and treated for the STI (*n* = 41), his potential to reinfect others (*n* = 38), and his potential to reinfect the participant (*n* = 33).

To identify any differences in partner notification and treatment preferences after testing positive for an STI, participants were requestioned at follow-up about their preferences for partner notification. Again, most participants replied that they would give their partners items provided by the clinic (Table 4). Most participants (*n* = 32) reported a preference for accompanying their partner to the clinic for STI notification and treatment, while fewer preferred to tell him to get tested and treated (*n* = 15), to give him pills (*n* = 6), or to give him a prescription for antibiotics (*n* = 1).

Nine participants in the follow-up study completed the interview via telephone and did not return to the clinic to provide a urine sample for STI testing. Of the 46 participants in the follow-up study with STI data, 11 were diagnosed with a repeat STI (23.9%). Seven participants had Chlamydia and four had gonorrhea; none were coinfected with both STIs. Four of the participants had discordant STI results at baseline and follow-up in that they were initially diagnosed with Chlamydia but then tested positive for gonorrhea at follow-up. The remaining seven cases were diagnosed with Chlamydia at both time points.

## 4. Discussion and Conclusion

STI rates at baseline (26.7%) and repeat infection after three months (23.9%) were high in this population of sexually active, African-American, adolescent women attending a public clinic for care. Four of the repeat cases consisted of a different STI diagnosis between baseline and follow-up. The remaining seven cases could be reinfections, but this status cannot be confirmed without knowledge of their partner's treatment status and behavior. Although high, the proportion of participants with repeat STI is consistent with other reports: a recent systematic review found reinfection rates of Chlamydia to range from 0% to 32% with a median of 13.9% for all age groups and reinfection rates of gonorrhea to range from 2.6% to 40% with a median of 11.7% [12]. Other studies conducted among adolescent females have reported recurrent infection rates as high as 40%–57% [13–15]. Chlamydia and gonorrhea are associated with adverse outcomes, including pelvic inflammatory disease and ectopic pregnancy, and additional infections can lead to worse outcomes [16]. To reduce long-term, adverse reproductive health outcomes, such as infertility, we should aim to reduce STI reinfection, especially among high-risk populations. Two possible approaches are to improve the likelihood that adolescents will inform their partner of their diagnosis and that these partners will receive treatment.

The American College of Obstetricians and Gynecologists and CDC have endorsed EPT as a practical alternative to traditional partner referral [17, 18]. Although EPT has been shown to reduce repeat Chlamydia and gonorrhea infections [19, 20], barriers and concerns regarding its implementation remain [4, 17, 21]. EPT is not universally available in the USA and is explicitly legal in only 35 states [22]. In Georgia, where the study was conducted, EPT is neither explicitly allowed nor prohibited. Interestingly, the high-risk population in the present study did not indicate a preference for EPT. Most adolescents reported that they could and would give their most recent sexual partner medication or a prescription if provided by the clinic; however, most reported preferring to bring their partner to the clinic over EPT or patient referral. A prior study on the acceptability of hypothetical options for treating exposed partners found high acceptance of EPT among adolescents, but the investigators did not include the option of bringing partners to the clinic [23]. The present study suggests that reproductive health clinics treating adolescent women should offer appointments for their partners. At Title X-funded clinics, male reproductive health care is encouraged; however, at other clinics, treating male partners could require major changes, such as hiring additional staff or additional training of primary health care providers (typically gynecologists and nurse practitioners).

As with other management schemes, the EPT use does not eliminate the need for partner notification. Male partner notification could be as important as screening women in order to decrease reinfection rates of Chlamydia. A recent modeling analysis estimated that increasing either Chlamydia screening by 3-fold or partner notification by 2-fold in Region X (Alaska, Idaho, Oregon, and Washington) in the USA could cause a 23% reduction in positivity [24].

Participants who reported notifying their partners about their positive STI diagnosis were more likely to have high self-efficacy for partner notification measured at baseline. Although the CI for this association was wide, the finding was statistically significant, is plausible, and is consistent with previous studies demonstrating a positive correlation between self-efficacy and partner notification among adolescent women [9] and adult men and women [25]. A larger study would be needed to confirm the finding in the study population.

High self-efficacy for partner notification of a positive STI was associated with older age and having fewer lifetime sexual partners. Thus, one approach could be for providers to target counseling and interventions for increasing partner notification to younger patients and those with more lifetime partners. Counseling techniques may include practicing the conversation with a health care provider, and interventions could focus on providing a safe place within the clinic to bring their partners and increasing support from health care providers. The present findings, though, show that partner notification is complicated by women's concerns about or experiences with negative reactions from their partner, including fears of physical violence. Previous research has found association between experiencing intimate partner violence and STI risk among female adolescents [26, 27]. Thus, counseling should include questions about the potential for partner violence in order to help women prepare to safely inform their partner of the positive diagnosis. Alternative options (e.g., provider or internet-based notification) could be considered.

Study limitations included low enrollment into the follow-up study. Of the 93 adolescents who had a positive STI diagnosis at baseline, 47 were lost to attrition or only answered the follow-up questionnaire by telephone and, consequently, did not have follow-up STI testing performed. Many participants who tested positive for an STI never returned to the clinic after their baseline or treatment visit. If the 47 women were uniformly negative for STI at follow-up, the true repeat reinfection rate could have been as low as 11.8%. Conversely, if they were all positive after three months, the repeat rate would have been 62.4%, which would be much higher than the national reinfection rates for African-American females but closer to the rates (4%–57%) in other studies of adolescent women [13–15]. Another limitation was the study reliance on self-reported data. ACASI was used in an attempt to decrease interviewer bias and to improve the likelihood of collecting valid reports, but partners were not contacted to confirm notification or treatment. Finally, our findings may not be generalizable to populations other than adolescent, African-American females who have access to Title X clinics or other similar sliding-scale or free reproductive health clinics.

This study provides important information regarding preferences for partner notification and treatment strategies among African-American, adolescent women attending an urban clinic in the southern USA both before and after a recent STI diagnosis. Our findings may help tailor future counseling and intervention approaches to adolescent women who are less likely to notify their partners of a positive STI diagnosis and therefore are at higher risk for STI reinfection. The present study population might benefit from clinic-based interventions, such as increasing the number of clinics that provide care to both males and females, offering EPT, and establishing concurrent treatment appointments for adolescent women to bring their partners with them for care.

## Figures and Tables

**Table 1:** Baseline demographic, reproductive history and behavioral characteristics, overall and by sexually transmitted infection status at baseline.

	Overall		STI-negative^a^		STI-positive^a^		
Characteristic	(*N* = 350)		(*N* = 255)		(*N* = 93)		*P* value^b^
	Number	(%)	Number	(%)	Number	(%)	
Age							
14–16	122	(34.9)	82	(32.2)	40	(43.0)	0.06
17–19	228	(65.1)	173	(67.8)	53	(57.0)	
Any health insurance							
No or do not know	134	(38.3)	97	(38.0)	35	(37.6)	0.95
Yes	216	(61.7)	158	(62.0)	58	(62.4)	
Ever seen in clinic							
No	119	(34.0)	74	(29.0)	44	(47.3)	<0.01
Yes	231	(66.0)	181	(71.0)	49	(52.7)	
Reason for clinic visit							
Pregnancy test	130	(37.1)	87	(34.1)	41	(44.1)	0.09
Birth control	183	(52.3)	138	(54.1)	45	(48.4)	0.34
STI testing	149	(42.6)	104	(40.8)	45	(48.4)	0.20
Think might have an STI							
No	255	(72.9)	198	(77.7)	55	(59.1)	<0.01
Yes or maybe	95	(27.1)	57	(22.4)	38	(40.9)	
Ever told had an STI							
No	197	(56.3)	149	(58.4)	46	(49.5)	0.14
Yes	153	(43.7)	106	(41.6)	47	(50.5)	
Ever pregnant							
No	258	(73.7)	188	(73.7)	68	(73.1)	0.91
Yes	92	(26.3)	67	(26.3)	25	(26.9)	
Age at first sex							
<15	150	(42.9)	106	(41.6)	44	(47.3)	0.34
≥15	200	(57.1)	149	(58.4)	49	(52.7)	
Lifetime sex partners							
1–3	189	(54.0)	140	(54.9)	47	(50.5)	0.47
4–7	161	(46.0)	115	(45.1)	46	(49.5)	
Description of most recent partner							
Serious boyfriend	176	(50.3)	131	(51.4)	43	(46.2)	0.17
On and off boyfriend	49	(14)	31	(12.2)	18	(19.4)	
Friend	30	(11.4)	19	(7.5)	11	(11.8)	
No one special	8	(3.0)	5	(2.0)	3	(3.2)	
Other/missing	87	(24.9)	69	(27.1)	18	(19.4)	
Agreed to monogamy with most recent sex partner							
No	58	(16.7)	41	(16.1)	17	(18.5)	0.61
Yes	290	(83.3)	213	(83.9)	75	(81.5)	

STI = sexually transmitted infection.

^a^Missing STI diagnosis, *n* = 2.

^b^From chi-squared test of difference by STI status.

**Table 2:** Baseline participant preferences for partner notification, overall and by sexually transmitted infection status at baseline.

	Overall		STI-negative		STI-positive		
Preferences	(*N* = 350)		(*N* = 255)		(*N* = 93)		*P* value^a^
	Number	(%)	Number	(%)	Number	(%)	
Preferred method for informing partner of need							
to be tested and treated for STI							
Participant informs him	297	(85.1)	221	(86.7)	75	(81.5)	0.46
Provider informs him	47	(13.5)	31	(12.2)	15	(16.3)	
Would not want him told	5	(1.4)	3	(1.2)	2	(2.2)	
Preferred method for STI treatment for partner							
Accompany him to clinic	247	(71.0)	180	(71.2)	65	(70.7)	0.52
Tell him to get tested and treated	61	(17.6)	47	(18.6)	14	(15.2)	
Give him pills or prescription	39	(11.2)	26	(10.3)	13	(14.1)	
If clinic provided item, would give to partner							
Antibiotic prescription	327	(93.7)	240	(94.1)	85	(92.4)	0.56
Antibiotic pills	336	(96.3)	246	(96.5)	88	(95.7)	0.72
Referral sheet	336	(96.6)	245	(96.5)	89	(96.7)	0.90
Pamphlet about her STI	328	(94.3)	241	(94.9)	85	(92.4)	0.38
Clinic appointment for partner	337	(96.8)	246	(96.9)	89	(96.7)	0.96

STI = sexually transmitted infection.

^a^From chi-squared test of difference by STI status.

**Table 3:** Baseline correlates of high self-efficacy for partner notification (*N* = 350).

Correlate	High self-efficacy		Bivariable analysis		Multivariable analysis^a^	
	Number	(%)	OR	(95% CI)	OR	(95% CI)
Age						
14–16 (*n* = 122)	70	(57.4)	1.0		1.0	
17–19 (*n* = 228)	162	(71.1)	1.8	(1.2, 2.9)	2.0	(1.3, 3.3)
Ever told had an STI						
No (*n* = 197)	127	(64.5)	1.0			
Yes (*n* = 153)	105	(68.6)	1.2	(0.8, 1.9)		
Ever pregnant with current partner						
No (*n* = 303)	192	(63.4)	1.0		1.0	
Yes (*n* = 47)	40	(85.1)	3.3	(1.4, 7.6)	2.3	(0.9, 5.4)
Age at first sex						
<15 (*n* = 150)	94	(62.7)	1.0			
≥15 (*n* = 200)	138	(69.0)	1.3	(0.8, 2.1)		
Lifetime number of sex partners						
1–3 (*n* = 189)	139	(73.5)	2.0	(1.3, 3.2)	2.0	(1.2, 3.2)
4–7 (*n* = 161)	93	(57.8)	1.0		1.0	
Relationship length with most recent sex partner						
No longer partner/≤6 months (*n* = 191)	122	(63.9)	1.0			
>6 months (*n* = 159)	110	(69.2)	1.3	(0.8, 2.0)		
Most recent sex partner was a “serious boyfriend”						
No (*n* = 174)	103	(59.2)	1.0		1.0	
Yes (*n* = 176)	129	(73.3)	1.9	(1.2, 3.0)	1.3	(0.8, 2.2)
Agreed to monogamy with most recent sex partner						
No (*n* = 58)	30	(51.7)	1.0		1.0	
Yes (*n* = 290)	201	(69.3)	2.1	(1.2, 3.7)	1.6	(0.8, 3.0)

CI = confidence interval; OR = odds ratio; STI = sexually transmitted infection.

^a^Adjusted for all variables in column with data.

**Table 4:** Experiences and preferences for partner notification, follow-up study (*N* = 55).

Experience and preference	Number	(%)
*Informed partner of STI diagnosis *		
No	13	(23.6)
Yes	42	(76.4)
Partner's reaction^a^		
Accepted the news well	19	(45.2)
Became upset	11	(26.2)
Accused participant of having sex with another	10	(23.8)
Threatened participant with physical abuse	1	(2.4)
Did not believe participant	5	(11.9)
Responded that already he was aware of it	6	(14.3)
Asked participant questions to learn more	17	(40.5)
Information provided to partner^a^		
Participant was STI positive	42	(100.0)
Name of STI	37	(88.1)
Name of medicine he should take	17	(40.5)
His need to be tested	41	(97.6)
His need for treatment	41	(97.6)
Location for his testing and treatment	32	(76.2)
Reason why it is important to be treated	37	(88.1)
Potential to reinfect participant	33	(78.6)
Potential to reinfect others	38	(90.5)
Need to abstain from sex for 7 days after both being treated	30	(71.4)
*If provided, would give to* *partner* ^a^		
Prescription	49	(89.1)
Pills	49	(89.1)
Referral sheet	50	(90.9)
Information pamphlet	48	(87.3)
Appointment in clinic	50	(90.9)
*If given choice, preference for partner notification and treatment for an STI *		
Accompany him to clinic	32	(58.2)
Tell him to get tested/treated	15	(27.3)
Give him pills	6	(10.9)
Give him prescription	1	(1.8)

STI = sexually transmitted infection.

^a^Multiple responses possible.
